# Selection of suitable reference lncRNAs for gene expression analysis in *Osmanthus fragrans* under abiotic stresses, hormone treatments, and metal ion treatments

**DOI:** 10.3389/fpls.2024.1492854

**Published:** 2025-01-21

**Authors:** Yingting Zhang, Qingyu Yan, Hui Xia, Xiangling Zeng, Jie Yang, Xuan Cai, Zeqing Li, Hongguo Chen, Jingjing Zou

**Affiliations:** ^1^ National Forestry and Grassland Administration Engineering Research Center for Osmanthus fragrans, Hubei University of Science and Technology, Xianning, China; ^2^ Osmanthus Innovation Center of National Engineering Research Center for Floriculture, Hubei University of Science and Technology, Xianning, China; ^3^ College of Forestry, Central South University of Forestry and Technology, Changsha, China; ^4^ Research Center for Osmanthus fragrans, Xianning Research Academy of Industrial Technology of Osmanthus fragrans, Xianning, China

**Keywords:** *Osmanthus fragrans*, reference lncRNAs, flowering stage, abiotic stress, hormone treatment, metal ion treatment

## Abstract

**Intoduction:**

*Osmanthus fragrans*, a well-regarded traditional flower in China, holds extensive applications in gardening, food, cosmetics, and traditional Chinese medicine. Despite its importance, research on long non-coding RNAs (lncRNAs) in *O. fragrans* has been constrained by the absence of reliable reference genes (RGs).

**Methods:**

We employed five distinct algorithms, i.e., delta-Ct, NormFinder, geNorm, BestKeeper, and RefFinder, to evaluate the expression stability of 17 candidate RGs across various experimental conditions.

**Results and discussion:**

The results indicated the most stable RG combinations under different conditions as follows: cold stress: lnc00249739 and lnc00042194; drought stress: lnc00042194 and lnc00174850; salt stress: lnc00239991 and lnc00042194; abiotic stress: lnc00239991, lnc00042194, lnc00067193, and lnc00265419; ABA treatment: lnc00239991 and *18S*; MeJA treatment: lnc00265419 and lnc00249739; ethephon treatment: lnc00229717 and lnc00044331; hormone treatments: lnc00265419 and lnc00239991; Al^3+^ treatment: lnc00087780 and lnc00265419; Cu^2+^ treatment: lnc00067193 and *18S*; Fe^2+^ treatment: lnc00229717 and *ACT7*; metal ion treatment: lnc00239991 and lnc00067193; flowering stage: lnc00229717 and *RAN1*; different tissues: lnc00239991, lnc00042194, lnc00067193, *TUA5*, *UBQ4*, and *RAN1*; and across all samples: lnc00239991, lnc00042194, lnc00265419 and *UBQ4*. The reliability of these selected RGs was further validated by analyzing the expression patterns of lnc00003036, lnc00126603, and lnc00250780. This study represents the first comprehensive evaluation of lncRNA RG stability in *O. fragrans*, significantly enhancing the accuracy and depth of lncRNA expression analyses in this species, contributing to advancements in plant stress resistance breeding and improving environmental adaptability.

## 1 Introduction

Sweet osmanthus (*Osmanthus fragrans*), a member of the Oleaceae family, is renowned as one of the ten most famous traditional flowers in China. It is highly valued for landscaping and is extensively utilized in food, cosmetics, and traditional Chinese medicine (Fu et al., 2022; Shi et al., 2024; Zhou et al., 2024). Despite its adaptability to various environmental conditions, *O. fragrans* is increasingly subjected to abiotic stresses, such as extreme temperatures, drought, and salinity (Li et al., 2020; Zhu et al., 2022; Yue et al., 2024b), and metal ion pollution, all exacerbated by global climate change and environmental degradation (Dang et al., 2022, 2024; Yue et al., 2024a). To address these challenges, it is crucial to investigate the molecular mechanisms underlying plant responses to these stress conditions. Currently, molecular biology research on *O. fragrans* predominantly focuses on functional gene analysis to elucidate its growth, development, and resistance mechanisms. However, no studies have yet been reported on long non-coding RNAs (lncRNAs) in this species.

LncRNAs are a class of RNA molecules longer than 200 nucleotides that do not encode proteins. Recently, lncRNAs have garnered considerable attention for their crucial roles in regulating gene expression (Prabakaran, 2016; De et al., 2024). Unlike traditional protein-coding genes, lncRNAs regulate gene expression through various mechanisms, including the modulation of chromatin structure, transcription, RNA processing, and translation (Bridges et al., 2021; Cui, 2023). In plants, lncRNAs are essential for developmental regulation (Li et al., 2015) and stress responses (Wu et al., 2019; Jia et al., 2023; Jin et al., 2024), encompassing responses to abiotic stress, pest and disease defense, and hormonal regulation.

Gene expression analysis is a widely used technique for investigating gene function, providing insights into the molecular processes that govern plant organ development and stress resilience. Techniques such as microarray, northern blotting, semiquantitative reverse transcription-PCR (RT-PCR), and quantitative reverse transcription-PCR (qRT-PCR) are commonly employed in these studies. Among these, qRT-PCR is particularly noteworthy for its high sensitivity, specificity, speed, and throughput, making it an essential tool for the relative quantification of gene expression (Hu et al., 2009; Zhang et al., 2018; Ni et al., 2019). The accuracy of qRT-PCR depends heavily on the stability of housekeeping genes used for data normalization. Key factors influencing this accuracy include primer specificity, RNA quality and integrity, reverse transcription efficiency, amplification efficiency (E value), and the quantity of initial material (Klein, 2002; Fleige and Pfaffl, 2006). Therefore, selecting stably expressed housekeeping genes as reference genes (RGs) is essential for error correction and standardization.

Typically, genes involved in fundamental cellular functions are selected as RGs, including 18S rRNA (*18S*) (Zhang et al., 2021b), actin (*ACT*) (Liu et al., 2016; Zhang et al., 2021b), cyclophilin (*CYP*) (Li et al., 2024), elongation factor 1 beta (*EF1B*) (Liu et al., 2016), glyceraldehyde-3-phosphate dehydrogenase (*GAPDH*) (Wang et al., 2024a), ras-related nuclear protein 1 (*RAN1*), α-tubulin (*TUA*) (Li et al., 2024), and ubiquitin (*UBQ*) (Tang et al., 2023). However, increasing experimental evidence indicates that RGs may not be as stable as previously believed under various conditions, and ideal RGs consistently expressed across all experimental conditions are rare (Jain et al., 2006; Løvdal and Lillo, 2009). The stability of RG expression can vary significantly across different species, stresses, and tissues. For example, *CYP*, *ACT*, *UBC*, and *18S* are reliable for studying gene expression in Chinese cedar (*Cryptomeria fortunei*) under abiotic stress, hormone treatments, and various tissues (Zhang et al., 2021b). In contrast, *ACT* exhibits high instability in wild barley (*Hordeum brevisubulatum*) under diverse stress treatments, including low and high temperatures, salt, drought, abscisic acid (ABA), gibberellic acid 3 (GA_3_), and ethephon (Zhang et al., 2018). Similarly, *18S* is the most unstable RG in hardy rubber tree (*Eucommia ulmoides*) under abiotic stress and across various tissues (Ye et al., 2018), and *CYP2* exhibits high instability in switchgrass (*Panicum virgatum*) under cadmium, mercury, chromium, and arsenic stresses (Zhao et al., 2020). Therefore, evaluating the expression stability of RGs under specific experimental conditions is essential before their application. Several statistical algorithms, including delta-Ct (Silver et al., 2006), geNorm (*v*3.5) (Vandesompele et al., 2002), NormFinder (*v*0.953) (Andersen et al., 2004), BestKeeper (*v*1.0) (Pfaffl et al., 2004), and Refinder, have been developed to assess and rank the stability of candidate RGs. However, these methods have limitations in terms of applicability and accuracy, particularly for lncRNA expression studies. Since lncRNA expression levels are generally lower than those of mRNA (Wu et al., 2014; Chen et al., 2018), using protein-coding genes as internal RGs may not accurately reflect lncRNA expression levels. To date, no systematic evaluation of RGs for lncRNA studies in *O. fragrans* has been reported. This gap highlights the necessity of this study to identify reliable RGs for accurate lncRNA expression analysis in *O. fragrans*.

In the initial stages of our experiment, our research team performed high-throughput sequencing of lncRNAs during the flower opening and senescence stages of *O. fragrans*. Using the fragments per kilobase of transcript per million mapped reads (FPKM) method to calculate expression levels, we selected 10 highly expressed and stable lncRNAs as candidate RGs (**Supplementary Table S1**), along with 7 common used internal RGs. We then employed qRT-PCR to analyze the expression stability of these lncRNAs under various conditions, including abiotic stress (low temperature, drought, and salt), hormone treatments (ABA, methyl jasmonate (MeJA), and ethephon), metal ion treatments (Fe^2+^, Al^3+^, and Cu^2+^), different tissues (stem, root, seed, leaf, and flower), and during flower opening and senescence stages. This analysis utilized the delta-Ct, geNorm, NormFinder, BestKeeper, and RefFinder systems, and identified appropriate internal RGs for each condition, providing reliable RGs for quantifying lncRNA expression in *O. fragrans* under different environmental conditions. This study establishes a scientific foundation and technical support for research on stress resistance in *O. fragrans* and its applications. Additionally, these findings provide a reference for lncRNA functional studies in other plants, contributing to advancements in plant stress resistance breeding and improving environmental adaptability.

## 2 Materials and methods

### 2.1 Plant material and treatments

A pest-free and well-growing Chang’e tree from Xianning (Hubei, China) was selected as the mother tree. In May 2023, semi-lignified branches with 2–3 lateral buds, each 12–16 cm in length, were collected as cuttings. The cuttings were prepared with flat cuts at the upper end and 45°cuts at the lower end, then soaked in distilled water for 12 hours. They were surface-sterilized with 1% (*m*/*v*) calcium hypochlorite (Ca(ClO)_2_) for 10 minutes, rinsed three times with distilled water, and soaked in 0.1 g L^-1^ GGR rooting powder (Aibiti Biotechnology Co., Ltd., Beijing, China) for 4 hours. The treated cuttings were then transplanted into a mixed soil matrix comprising peat, perlite, vermiculite, and yellow sand (1/1/1/1, *v*/*v*/*v*/*v*) and subsequently moved to the *O. fragrans* base in Xianning, Hubei, China.

In March 2024, 120 uniformly growing *O. fragrans* plants, each measuring 0.5–0.6 m in height, were selected and planted on the campus of Hubei University of Science and Technology (114°19’52’’E, 29°51’19’’N) for seedling acclimatization. In April 2024, 90 of these plants, exhibiting similar growth status, were selected for nine different stress tests. For abiotic stress tests, the cuttings were exposed to 4°C to simulate low-temperature stress; the cuttings were sprayed with 300 mM NaCl and 20% PEG-6000 to induce salt and drought stress, respectively. For hormone treatments, the plants were sprayed evenly with 300 μM ABA, 300 μM MeJA, and 5 mM ethephon, respectively. For metal ion treatments, the cuttings received sprays of 3 mM CuSO_4_·5H_2_O, 3 mM AlCl_3_·6H_2_O, and 3 mM FeSO_4_, respectively. Each treatment involved the application of 200 mL per plant to ensure complete coverage of all leaves. Except for the low-temperature stress group, all seedlings were kept in a controlled environment chamber at 25°C with a 12-hour light/12-hour dark photoperiod and 60% humidity. Three plants were used for each treatment, and each treatment was replicated three times (3 × 3 plants). Samples were collected at 0, 3, 6, 12, 24, and 72 hours after treatment. Additionally, samples from different tissues of *O. fragrans*, including roots, stems, leaves, seeds, and flowers, were collected, flash-frozen in liquid nitrogen, and stored at –80°C.

Additionally, a healthy and pest-free *O. fragrans* plant from Huazhong Agricultural University (114°21’W, 30°29’N) was utilized for flower tissue collection. Samples were collected at 10 am from five developmental stages: S1 (bud stage), S2 (early flowering stage), S4 (full flowering stage), S5 (late flowering stage), and S6 (petal shedding stage). For each developmental stage, three biological replicates were prepared. The samples were rapidly frozen in liquid nitrogen and stored at –80°C for subsequent analysis.

### 2.2 Extraction of total RNA and synthesis of first strand cDNA

Total RNA was extracted from each sample using the HiPure Plant RNA Mini Kit (Magen Biotechnology (Guangzhou) Co., Ltd., Guangzhou, Guangdong, China) following the manufacturer’s instructions. The integrity and concentration of the RNA were assessed using 1% agarose gel electrophoresis and a spectrophotometer (NanoDrop 2000, Thermo Scientific, Wilmington, DE, USA). Subsequently, 1 μg of RNA was reverse transcribed into cDNA using the HiScript^®^ III RT SuperMix for qPCR Kit (+gDNA wiper) (Vazyme Biotechnology Co., Ltd., Nanjing, Jiangsu Province, China). The resulting cDNA was stored at –20°C for subsequent analysis.

### 2.3 Identification of candidate RGs and primer design

Through a comprehensive reference review, common RGs such as *18S*, *ACT*, *EF1B*, *GAPDH*, *RAN1*, *TUA*, and *UBQ*, reported in other species, were compared with the whole genome data of *O. fragrans* using local BLAST (blastVer: 2.4.0+). This comparison aimed to identify candidate RGs with high homology. Additionally, based on RNA-seq data, 10 lncRNAs, i.e., lnc00031789, lnc00042194, lnc00044331, lnc00067193, lnc00087780, lnc00174850, lnc00229717, lnc00239991, lnc00249739 and lnc00265419, exhibiting relatively high expression levels (FPKM_max_ > 5) and fold changes < 1.4, were selected as potential RGs (**Supplementary Table S1**).

Primer design for qRT-PCR was performed using Primer Premier 5.0 (Premier Biosoft International, Palo Alto, CA, USA) with the following parameters: PCR product length of 70–300 bp, melting temperature of 58–62°C, and GC content of 40–60%. Primer specificity was verified using e-PCR (https://yanglab.hzau.edu.cn/OfIR/tools/epcr/). All primers listed in **Table 1** were synthesized by Tsingke Biotech Co., Ltd. (Nanjing, Jiangsu, China).

**Table 1:** Sequence and primer information for seventeen RGs and three target lncRNAs.

Gene	Gene Name	Registered Number/Gene Number	Primer Sequence (5’ to 3’)	AS (bp)	E (%)	R^2^
*18S*	18S ribosomal RNA	Chr10:28859089-28860895	CCATAAACGATGCCGACCAG	108	109.888	0.998
			GCCTTGCGACCATACTCCC			
*ACT7*	actin 7	LYG014821	AAATCACTGCCTTGGCTCCTA	176	99.775	0.997
			GCACTTCCTGTGGACGATAGA			
*EF1B*	eukaryotic translation elongation factor 1 beta	LYG015343	ACCTGGTTGGGTCTTCTATTCA	149	105.194	0.994
			AAACTCGGGAGTCTTGTTGGA			
*GAPH*	glyceraldehyde-3-phosphate dehydrogenase	LYG039872	GCTGCCATCAAGGAGGAGT	94	105.293	0.998
			GGCTATCACCCACAAAGTCG			
*RAN1*	ras-related nuclear protein 1	LYG033166	AGAACCGACAGGTGAAGGCAA	117	108.479	1.000
			TGGCAAGGTACAGAAAGGGCT			
*TUA5*	tubulin alpha 5	LYG023230	ATCATCGCTGACCACTTCTTTG	237	96.096	0.979
			GCCATGTATTTCCCGTGTCTT			
*UBQ4*	ubiquitin 4	LYG013775	ACTGCACCCTCCATTTGGT	165	98.346	0.992
			TGCCGTTCACGATTAGTTCTC			
lnc00031789	–	Chr11:14271087-14271761	AAAAGCAAGGCTGGTGAAGA	289	109.098	0.973
			GTTCGGGTAGAAGGTTTGAGTAG			
lnc00042194	–	Chr12:763266-765659	TCGGCGAAGGGTGAGTAATG	72	109.424	1.000
			TGAAGACGACGACGGGATT			
lnc00044331	–	Chr12:24681772-24686386	GGGTTGTGGCGGGTAATTCATCTTG	213	109.481	0.997
			GGTAAGGGATGTAATAATGTGCTGA			
lnc00067193	–	Chr14:23490741-23494983	GCATCGGCGATTGTGAGA	94	102.447	0.998
			AAGCGAAGGTCCGTTTGG			
lnc00087780	–	Chr16:8481231-8485782	CGAACTGCCCTTATGGTTATTC	283	91.216	0.985
			CCTGGCGTAGATTCCTTGC			
lnc00174850	–	Chr3:26469404-26470460	GCTCCCTGTCTCGATATTCATAC	161	111.998	0.997
			ATCTGTCGTCAAGCGTTCCT			
lnc00229717	–	Chr7:12951973-12954983	CTTAGCCGCCCATCCCA	208	106.643	0.999
			CACCCGCATTATCCGTTGA			
lnc00239991	–	Chr8:14604763-14607670	TTTCTTGGTCGTGTCTTTAGCA	81	91.951	0.992
			CAAGTTGCGGGAGACGTTAT			
lnc00249739	–	Chr9:2882156-2884663	TGGACTTGGCTGACCCTTGA	168	108.806	0.998
			TTCCAATCTTGCGGACTGAC			
lnc00265419	–	unchr23:1482700-1483090	CATTATTGTTACGCCGACCAC	94	108.595	0.999
			GATCGTTTAGCCGCTCTTTCT			
Target genes						
lnc00003036	–	Chr1:21663530-21664636	CCTCATTCCACTAATCCGTTGT	190	94.444	0.988
			GCCTCTAGCTCTATAATCCACCC			
lnc00126603	–	Chr2:36954708-36955347	GCTCGCCGGAGATCAAA	261	106.576	0.996
			ATCAGGCAGAGGAGGCTTATT			
lnc00250780	–	Chr9:27183623-27185284	GTGCCGCTGTGAAGGGAG	162	95.285	0.976
			CAGTAACAGAGTCACCGAGGG			

AS, amplicon size; E, PCR Efficiency; R^2^, correlation coefficient.

### 2.4 Primer specificity and amplification efficiency assays

Primer specificity was assessed using RT-PCR with the Fast PCR Kit (Vazyme). The reaction mixture included 1 μL of each forward and reverse primer (10 μM), 1 μL of cDNA, 10 μL of 2 × Rapid Taq Master Mix, and 7 μL of ddH_2_O. The amplification program consisted of an initial denaturation at 95°C for 3 minutes, followed by 35 cycles of 95°C for 15 seconds, 58°C for 15 seconds, and 72°C for 20 seconds, with a final extension at 72°C for 5 minutes. The accuracy of the primers was subsequently verified using 2.0% (*w*/*v*) agarose gel electrophoresis.

Three microliters of cDNA template from each sample were mixed and serially diluted 5-fold (1:4, 1:24, 1:124, 1:624, 1:3124; cDNA:water, *v*:*v*). qRT-PCR was performed using Taq Pro Universal SYBR qPCR Master Mix (Vazyme). The 20 µL reaction mixture comprised 10 µL of 2 × Taq Pro Universal SYBR qPCR Master Mix, 2 µL of 5-fold serially diluted cDNA, 0.4 µL of each forward and reverse primer (10 µM), and 7.2 µL of ddH_2_O. qRT-PCR was conducted on a Tianlong Gentier 96E system (Tianlong Technology Co., Ltd., Xi’an, China) with the following thermal cycling program: initial denaturation at 95°C for 30 seconds, followed by 40 cycles of 95°C for 10 seconds and 60°C for 30 seconds. Melting curves were generated from 60–95°C immediately after qRT-PCR to detect primer dimerization and other amplification artifacts. A non-template control was included for each gene, with each reaction performed in triplicate biological and technical replicates. Correlation coefficients (R^2^) and E values were calculated based on the qRT-PCR results (Whistler et al., 2010).

### 2.5 Gene expression stability analysis

All samples were diluted 10-fold (1:9, cDNA:water, *v*:*v*) and subjected to qRT-PCR to determine the original quantification cycle (Ct) values. The expression stability of the internal RGs was assessed using four different algorithms: delta-Ct (Silver et al., 2006), geNorm (*v*3.5) (Vandesompele et al., 2002), NormFinder (*v*0.953) (Andersen et al., 2004), and BestKeeper (*v*1.0) (Pfaffl et al., 2004). For stability analysis, geNorm and NormFinder converted the Ct values into 2^-ΔCt^ (where ΔCt = Ct value − the minimum Ct value of each group), while BestKeeper used the LinRegPCR program to calculate the E value and the coefficient of variation (CV) along with the standard deviation (SD) based on the Ct values. Additionally, geNorm determined the optimal number of internal RGs by calculating the paired difference value V_n_/V_n+1_ between two consecutive normalization factors. The geometric mean ranking was calculated as the average ranking of each gene across the four algorithms for each treatment, in different tissues, or across all samples. Additionally, RefFinder (https://blooge.cn/RefFinder/) was utilized to comprehensively validate the results of the RG stability analysis.

### 2.6 Validation of RGs using qRT-PCR

To test the reliability of the identified RGs, the expression levels of three lncRNAs, i.e., lnc00003036, lnc00126603 and lnc00250780, were normalized under each experimental condition using both the most optimal and least stable RGs, applying the 2^-ΔΔCt^ method (Livak and Schmittgen, 2000). The primer pairs for amplification were designed as follows: lnc00003036: forward 5′-CCTCATTCCACTAATCCGTTGT-3′ and reverse 5′-GCCTCTAGCTCTATAATCCACCC-3′; lnc00126603: forward 5′-GCTCGCCGGAGATCAAA-3′ and reverse 5′-ATCAGGCAGAGGAGGCTTATT-3′; and lnc00250780: forward 5′-GTGCCGCTGTGAAGGGAG-3′ and reverse 5′-CAGTAACAGAGTCACCGAGGG-3′ (**Table 1**).

## 3 Results

### 3.1 Assessment of primer specificity and PCR amplification efficiency

**Figure 1:**
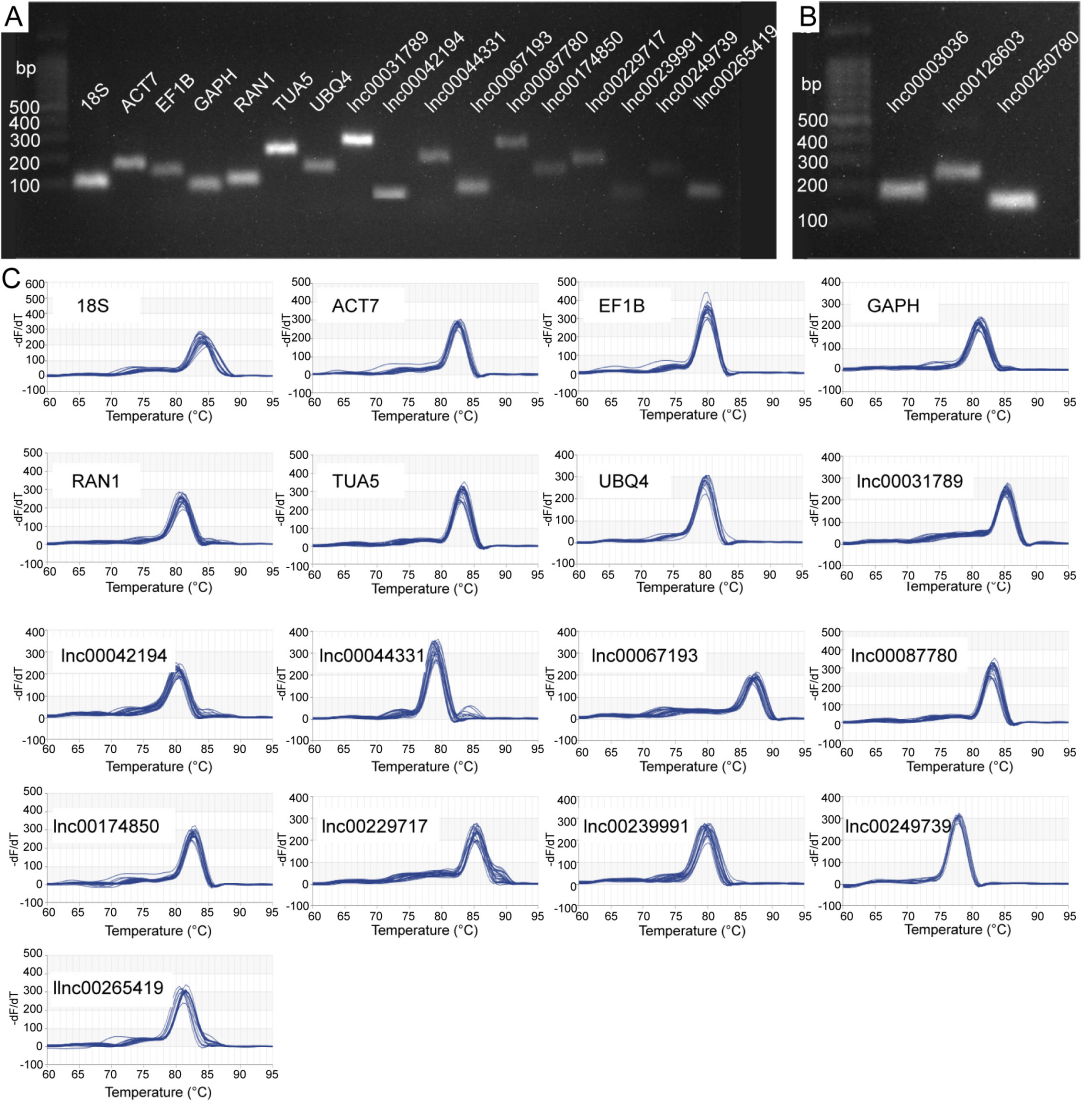
Specificity of candidate reference gene (RG) primer pairs. **(A, B)** 2.0% agarose gel electrophoresis. All primer pairs demonstrated specificity, with amplification products corresponding to the expected lengths. **(C)** Melting curves for the 17 candidate RGs. *18S*, 18S ribosomal RNA; *ACT7*, actin 7; *EF1B*, eukaryotic translation elongation factor 1 beta; *GAPH*, glyceraldehyde-3-phosphate dehydrogenase; *RAN1*, ras-related nuclear protein 1; *TUA5*, tubulin alpha-5; and *UBQ4*, ubiquitin-4.

We selected 17 candidate RGs for gene normalization studies, comprising 7 protein-coding genes and 10 lncRNAs (**Table 1**). To ensure primer specificity, we conducted 2.0% agarose gel electrophoresis and melting curve analysis. The electrophoresis results confirmed that each primer amplified a PCR product of the expected length, indicating appropriate primer design and high amplification specificity (**Figures 1A, B**). Melting curve analysis revealed a single melting peak for each primer (**Figure 1C**), indicating the absence of nonspecific amplification or primer dimer formation. The E values of all primers ranged from 91.216% (lnc00087780) to 111.998% (lnc00174850), with R^2^ values ranging from 0.973 (lnc00031789) to 1.000 (*RAN1* and lnc00042194) (**Table 1**). These results demonstrate that all 17 primer pairs meet the necessary criteria for qRT-PCR and are suitable for further analysis.

### 3.2 Expression profiling of candidate RGs

**Figure 2:**
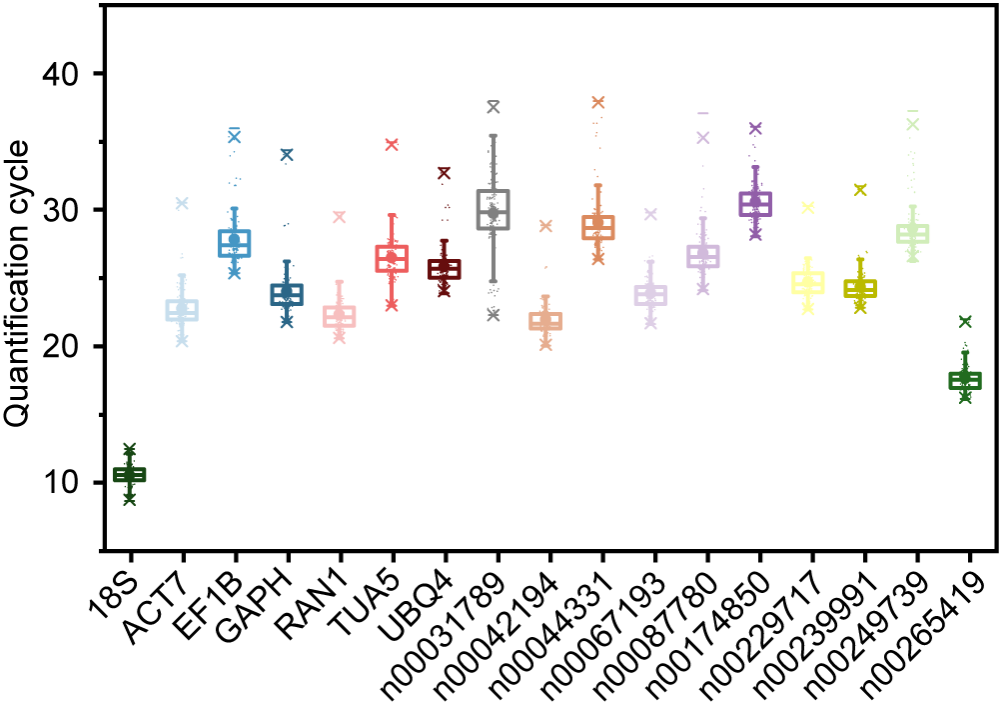
Quantification cycle (Ct) values of 17 candidate RGs under various experimental conditions. The boxes represent the interquartile range (25th to 75th percentiles) of Ct values, with the lines within the boxes indicating the medians. Whiskers extend to the 99% confidence intervals, and asterisks denote outliers. The upper and lower horizontal lines indicate the maximum and minimum values, respectively, and the small circles represent the average Ct values.

To evaluate the suitability of the 17 candidate RGs, we assessed their expression levels under various experimental conditions using qRT-PCR. The conditions included abiotic stresses (low temperature, drought, and salt), hormone treatments (ABA, MeJA, and ethephon), metal ion treatments (Fe^2+^, Al^3+^, and Cu^2+^), different tissues (stem, root, seed, leaf, and flower), as well as processes of flower opening and senescence. The Ct value, representing the cycle number at which the fluorescence signal exceeds the threshold, indicates the number of cycles required to detect a true signal from the sample. The average Ct values across all samples ranged from 10.556 (*18S*) to 30.580 (lnc00174850) (**Figure 2**). Notably, *18S* and lnc00265419 exhibited relatively low Ct values (**Figure 2**), indicating high initial copy numbers and elevated expression levels (Bustin et al., 2009). Conversely, genes such as lnc00249739, lnc00044331, lnc00174850, lnc00087780, lnc00031789, *TUA5*, *EF1B*, and *UBQ4* exhibited lower expression levels, as evidenced by their higher Ct values (**Figure 2**).

The Ct value range for *18S* was the smallest, at 3.820 Ct, with maximum and minimum values of 12.496 and 8.676, respectively (**Figure 2**), indicating minimal variation in expression across all samples. Lnc00265419 exhibited the second smallest Ct value range, at 5.896 Ct (**Figure 2**). In contrast, the expression level of lnc00031789 displayed the greatest variability, with a range of 15.664 Ct, and Ct values spanning from 22.301 to 37.965 (**Figure 2**). These results suggest that *18S* and lnc00265419 exhibit relatively stable expression levels across all samples, indicating their potential as stable RGs. However, further validation is required to confirm their suitability.

### 3.3 Expression stability of candidate RGs

The expression stability of candidate RGs was evaluated using four different algorithms: delta-Ct, geNorm, NormFinder, and BestKeeper, across various experimental conditions.

#### 3.3.1 Expression stability of candidate RGs assessed using the delta-Ct method

**Figure 3:**
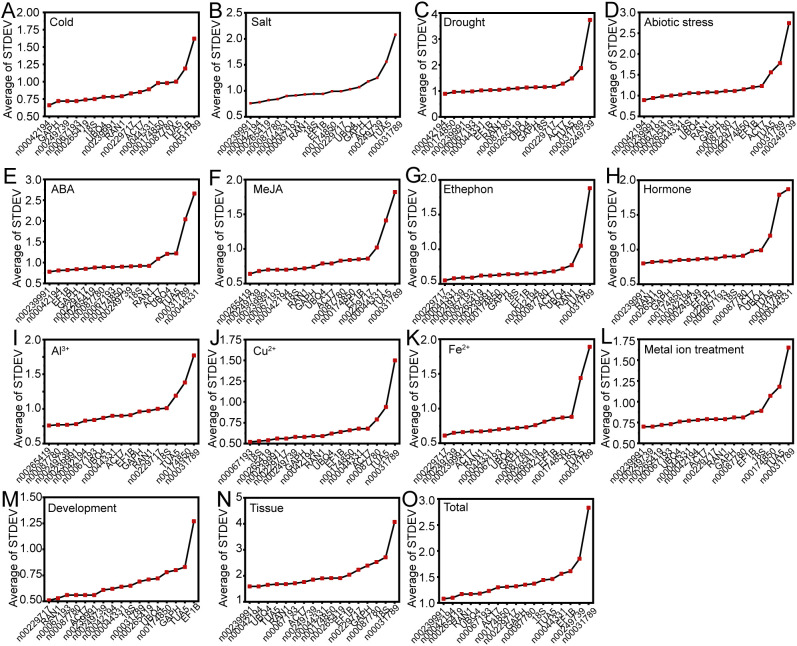
Mean standard deviation (STDEV) obtained through delta-Ct analysis. Panels display the STDEV for various experimental conditions: **(A)** cold, **(B)** salt, **(C)** drought, and **(D)** various abiotic stresses; **(E)** ABA, **(F)** MeJA, **(G)** ethephon, and **(H)** hormone treatments; **(I)** Al^3+^, **(J)** Cu^2+^, **(K)** Fe^2+^, and **(L)** metal ion treatments. **(M)** Flowering stage; **(N)** different tissues; and **(O)** across all samples.

The delta-Ct method was employed to evaluate the relative expression levels of RGs by analyzing the repeatability of the mean standard deviation (STDEV) of gene expression differences between samples (Silver et al., 2006). A smaller STDEV indicates greater stability in gene expression. Under conditions of low temperature, drought, and abiotic stress, lnc00042194 exhibited the most stable expression (**Figures 3A, C, D**). For salt stress, ABA treatment, hormone treatment, metal ion treatment, different tissues, and across all samples, lnc00239991 demonstrated greater stability compared to other internal RGs (**Figures 3B, E, H, L, N, O**). During ethephon treatment, Fe^2+^ treatment, and the flowering stage, lnc00229717 proved to be the most stable RG (**Figures 3G, K, M**). For MeJA, Cu^2+^, and Al^3+^ treatments, the most stable RGs were lnc00265419, lnc00067193, and lnc00265419, respectively (**Figures 3F, I, J**). Notably, lnc00031789 (14 instances, 93.33%) and *TUA5* (7 instances, 46.67%) were the least stable across most conditions (**Figure 3**; **Supplementary Table S2**).

#### 3.3.2 Expression stability of candidate RGs analyzed using GeNorm

**Figure 4:**
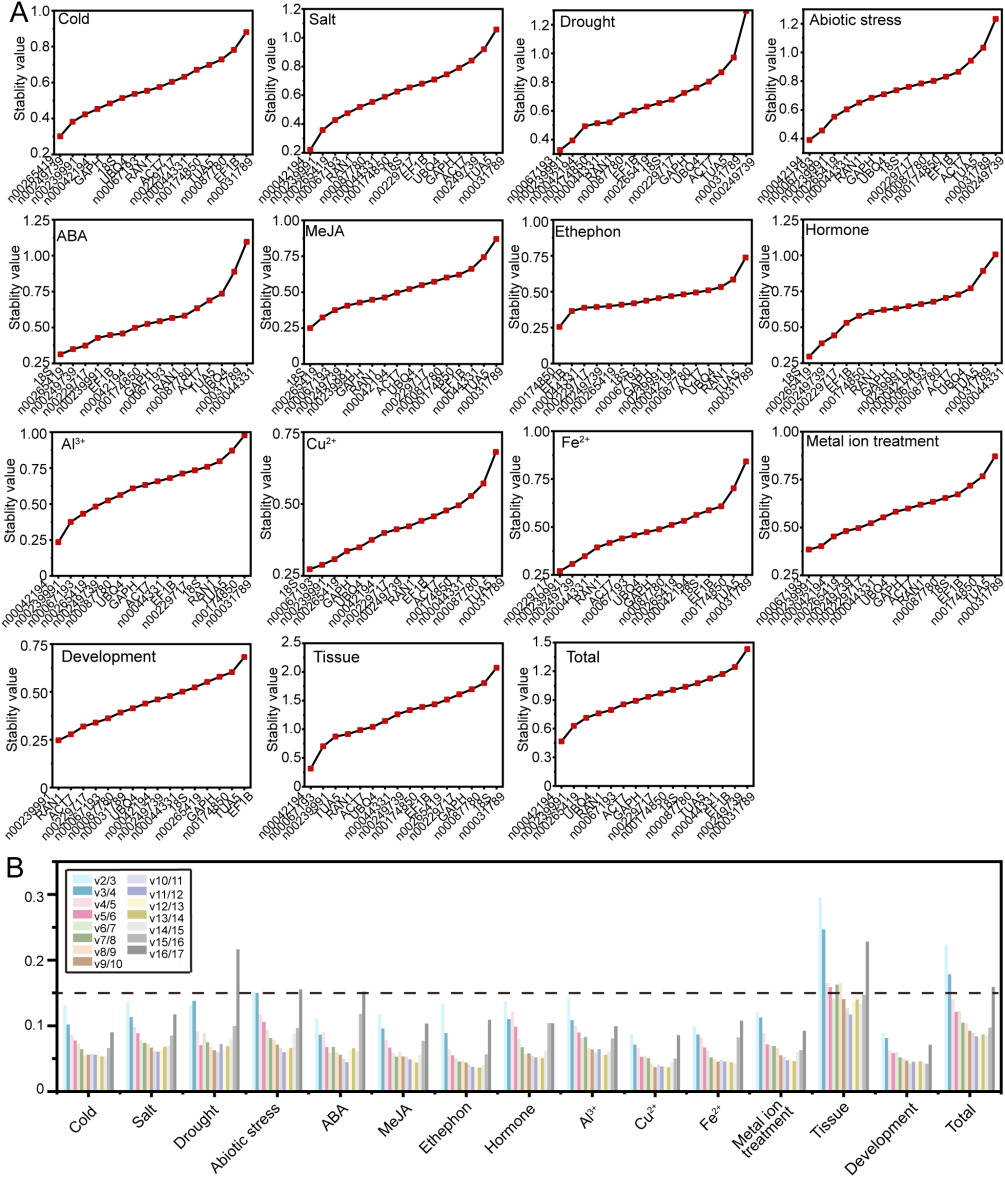
Expression stability and optimal number of RGs determined by GeNorm. **(A)** The stability values and rankings of the 17 candidate RGs across all treatments are presented, with lower M values indicating greater stability in gene expression. **(B)** The optimal number of RGs for accurate normalization in *O. fragrans* was determined by geNorm. The plot displays pairwise variation (V_n_/V_n+1_) values, with the optimal number of RGs identified based on the threshold of 0.15.

GeNorm evaluates RG stability by calculating the stability value (M value) based on the average pairwise variation (V value), with a lower M value indicating greater stability in gene expression (Vandesompele et al., 2002). The GeNorm analysis results, as shown in **Figure 4A**, reveal variability in RG stability across different experimental conditions. Specifically, lnc00042194 and lnc00239991 exhibited the greatest stability under salt stress, Al^3+^ treatment, and across all samples; lnc00067193 and lnc00239991 were the most stable under drought and metal ion stress; lnc00042194 and lnc00067193 demonstrated the highest stability under abiotic stress and in various tissues, while *18S* and lnc00265419 showed the highest stability during ABA and MeJA treatments. Additionally, specific gene pairs were identified as the most stable under particular conditions: lnc00265419 and lnc00249739 for low temperature, lnc00174850 and *EF1B* for ethephon treatment, *18S* and lnc00067193 for Cu^2+^ treatment, lnc00229717 and lnc00239991 for Fe^2+^ treatment, *18S* and lnc00265419 for hormone treatments, and lnc00239991 and *RAN1* for the flowering stage. Notably, in alignment with the delta-Ct analysis results, lnc00031789 (14 instances, 93.33%) and *TUA5* (7 instances, 46.67%) were consistently identified as the least stable across most conditions (**Figure 4A**, **Supplementary Table S2**).

In instances where a single RG may not provide sufficient stability for accurate normalization, multiple RGs may be required to enhance precision. GeNorm determines the optimal number of RGs by calculating pairwise variation (V_n_/V_n+1_) values, with a recommended threshold of 0.15 for optimal normalization. In this study, for different tissue samples, the V_6_/V_7_ value was 0.141, indicating that the most suitable combination of RGs included six genes: lnc00042194, lnc00067193, lnc00239991, *TUA5*, *RAN1*, and *ACT7* (**Figures 4A, B**). For all samples, the V_4_/V_5_ value was 0.141, suggesting that the optimal RG combination comprised four genes: lnc00042194, lnc00239991, lnc00265419, and *UBQ4* (**Figures 4A, B**). For abiotic stress, the V_4_/V_5_ value was also below 0.15, indicating that the best RG combination includes lnc00042194, lnc00067193, lnc00239991 and lnc00265419 (**Figures 4A, B**). For other treatments, the V_2_/V_3_ value was below 0.15, indicating that the most suitable RG combination consisted of two genes (**Figure 4B**).

#### 3.3.3 Expression stability of candidate RGs analyzed using NormFinder

**Figure 5:**
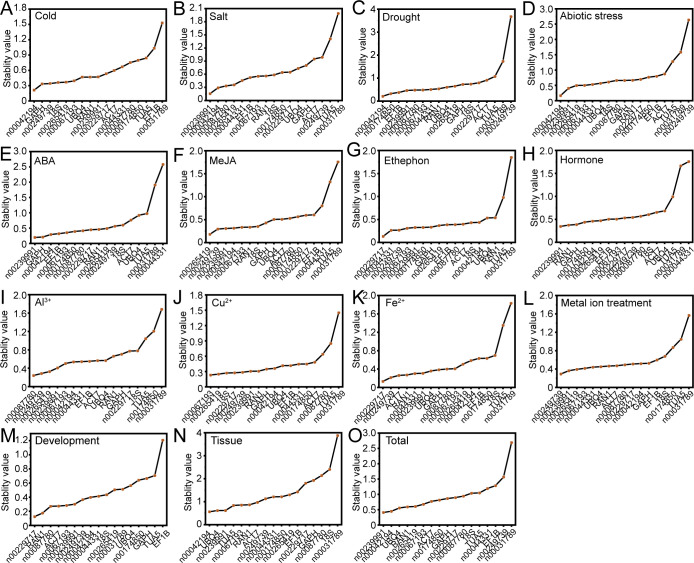
Expression stability of the candidate RGs as assessed by NormFinder. **(A)** Cold, **(B)** salt, **(C)** drought, and **(D)** various abiotic stresses; **(E)** ABA, **(F)** MeJA, **(G)** ethephon, and **(H)** hormone treatments; **(I)** Al^3+^, **(J)** Cu^2+^, **(K)** Fe^2+^, and **(L)** metal ion treatments; **(M)** flowering stage; **(N)** different tissues; and **(O)** across all samples.

The stability of candidate RGs was evaluated using the NormFinder program, which ranks genes based on their stability through ANOVA analysis (Andersen et al., 2004). As illustrated in **Figure 5**, lnc00239991 and lnc00042194 were identified as the most stable RGs under salt stress and across all tissues, consistent with the results from the geNorm analysis (**Figures 4A**, **5B, O**). However, NormFinder identified different optimal RGs for specific conditions compared to geNorm. For low temperature, drought, and various abiotic stresses, the most stable RG combinations were lnc00042194 and *GAPH*, lnc00042194 and lnc00174850, and lnc00239991 and lnc00042194, respectively (**Figures 5A, C, D**). For treatments involving ABA, MeJA, ethephon, and hormones, the most stable internal RG combinations were lnc00239991 and *GAPH*, lnc00265419 and lnc00249739, lnc00229717 and lnc00044331, and lnc00239991 and *RAN1*, respectively (**Figures 5E–H**). Under treatments with Cu^2+^, Fe^2+^, Al^3+^ and metal ions, the most stable RGs combinations were lnc00067193 and lnc00265419, lnc00229717 and lnc00249739, lnc00087780 and lnc00249739, and lnc00249739 and lnc00239991, respectively (**Figures 5I–L**). For different tissues and during the flowering stage, the most stable internal RGs were lnc00042194 and *UBQ4*, as well as lnc00229717 and *RAN1* (**Figures 5M, N**). Notably, in accordance with the delta-Ct and geNorm analyses, lnc00031789 (14 instances, 93.33%) and *TUA5* (7 instances, 46.67%) were the least stable across most conditions (**Figure 5**; **Supplementary Table S2**).

#### 3.3.4 Expression stability of candidate RGs analyzed using BestKeeper

**Figure 6:**
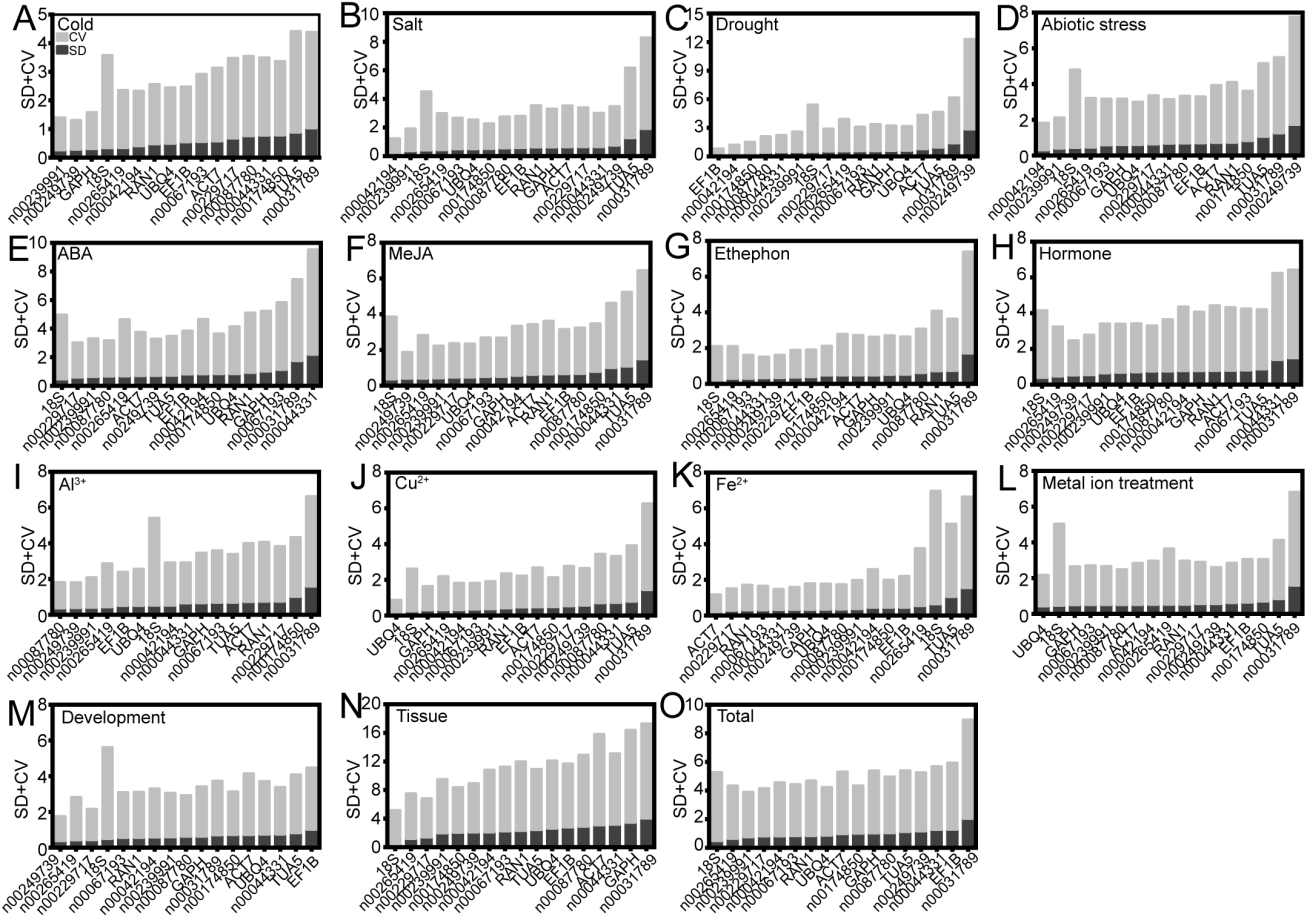
Expression stability of candidate RGs as calculated using BestKeeper under various conditions. These conditions include: **(A)** cold, **(B)** salt, **(C)** drought, and **(D)** various abiotic stresses; **(E)** ABA, **(F)** MeJA, **(G)** ethephon, and **(H)** hormone treatments; **(I)** Al^3+^, **(J)** Cu^2+^, **(K)** Fe^2+^, and **(L)** metal ion treatments; **(M)** flowering stage; **(N)** different tissues; and **(O)** across all samples.

BestKeeper evaluates the stability of RGs by calculating the SD and CV of Ct values, with RGs exhibiting an SD of less than 1.0 considered to be stably expressed (Pfaffl et al., 2004). A lower CV indicates higher stability of the RG. According to BestKeeper analysis, *18S* was identified as the most stable internal RG for ABA, MeJA, ethephon, hormone treatments, various tissues, and across all samples (**Figures 6E–H, N, O**). Under salt and abiotic stresses, lnc00042194 exhibited the highest stability (**Figures 6B, D**), whereas *UBQ4* was the most stable under Cu^2+^ and metal ion treatments (**Figures 6J, I**). The optimal internal RGs for low temperature, PEG, Fe^2+^ and Al^3+^ treatments, and the flowering stage were lnc00239991, *EF1B*, *ACT7*, lnc00087780, and lnc00249739, respectively (**Figures 6A, C, I, K, M**). Notably, consistent with the delta-Ct, geNorm, and NormFinder analyses, lnc00031789 (14 instances, 93.33%) and *TUA5* (8 instances, 53.33%) were identified as the least stable across most conditions (**Figure 6**; **Supplementary Table S2**).

### 3.4 Comprehensive analysis of expression stability of candidate RGs using RefFinder and the geometric mean method

**Figure 7:**
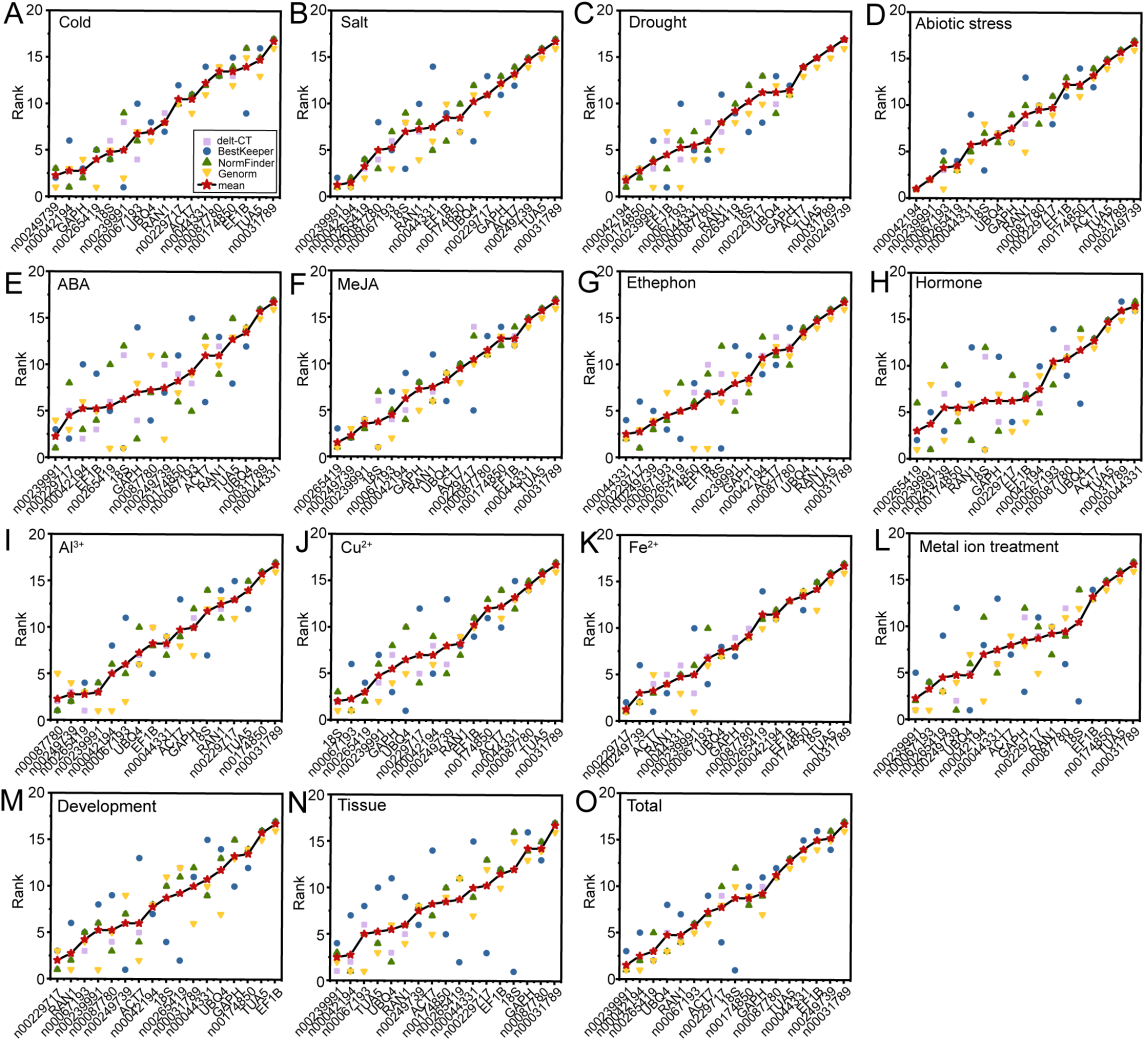
Comprehensive ranking of 17 RGs across all stress conditions. Rankings are calculated as the geometric mean of four ranking methods: delta-Ct, geNorm, NormFinder, and BestKeeper for each sample group. **(A)** Cold, **(B)** salt, **(C)** drought, and **(D)** various abiotic stresses; **(E)** ABA, **(F)** MeJA, **(G)** ethephon, and **(H)** hormone treatments; **(I)** Al^3+^, **(J)** Cu^2+^, **(K)** Fe^2+^, and **(L)** metal ion treatments; **(M)** flowering stage; **(N)** different tissues; and **(O)** across all samples.

The expression stability of 17 candidate RGs was comprehensively analyzed using four different algorithms. Each algorithm, based on distinct principles, produced varying stability rankings. To obtain a comprehensive ranking, we calculated the geometric mean of the rankings from delta-Ct, geNorm, NormFinder, and BestKeeper for each experimental condition. The most stable RG combinations under low-temperature stress, salt stress, drought stress, ABA, MeJA, ethephon, hormone treatments, Al^3+^, Cu^2+^, Fe^2+^, metal ion treatments, and flower opening and senescence stages were lnc00249739 and lnc00042194, lnc00239991 and lnc00042194, lnc00042194 and lnc00174850, lnc00239991 and lnc00229717, lnc00265419 and lnc00249739, lnc00229717 and lnc00044331, lnc00265419 and lnc00239991, lnc00087780 and lnc00249739, lnc00067193 and 18S, lnc00229717 and lnc00249739, lnc00239991 and lnc00067193, and lnc00229717 and *RAN1* (**Figures 7A–C, E–M**). For abiotic stress conditions, lnc00239991, lnc00042194, lnc00067193, and lnc00265419 were the most stable RGs (**Figure 7D**). In different tissues, lnc00239991, lnc00042194, lnc00067193, *TUA*, *UBQ4*, and *RAN1* were identified as the most stable RGs (**Figure 7N**). Across all samples, the optimal RG combinations were lnc00239991, lnc00042194, lnc00265419, and *UBQ4* (**Figure 7O**).

The results obtained from RefFinder (Xie et al., 2012, 2023) generally aligned with those derived using the geometric mean, with a few exceptions. Under ABA treatment, lnc00239991 and *18S* (instead of lnc00229717) were identified as the most stable RGs (**Figure 7E**; **Supplementary Table S3**). For Al^3+^ treatment, lnc00087780 and lnc00265419 (instead of lnc00249739) were the most stable (**Figure 7I**; **Supplementary Table S3**). In the case of Fe^2+^ treatment, lnc00229717 and *ACT7* (instead of lnc00249739) were determined to be the best RGs (**Figure 7K**; **Supplementary Table S3**).

### 3.5 Identification and validation of the most appropriate lncRNA RGs

To verify the accuracy of RG stability, we examined the relative expression levels of three target genes, i.e., lnc00003036, lnc00126603, and lnc00250780, under various experimental conditions. Both stable and unstable genes were used for normalization. These conditions examined included cold stress, salt stress, drought stress, ABA treatment, ethephon treatment, MeJA treatment, Al^3+^ treatment, Cu^2+^ treatment, Fe^2+^ treatment, flower opening and senescence, and various tissues.

**Figure 8:**
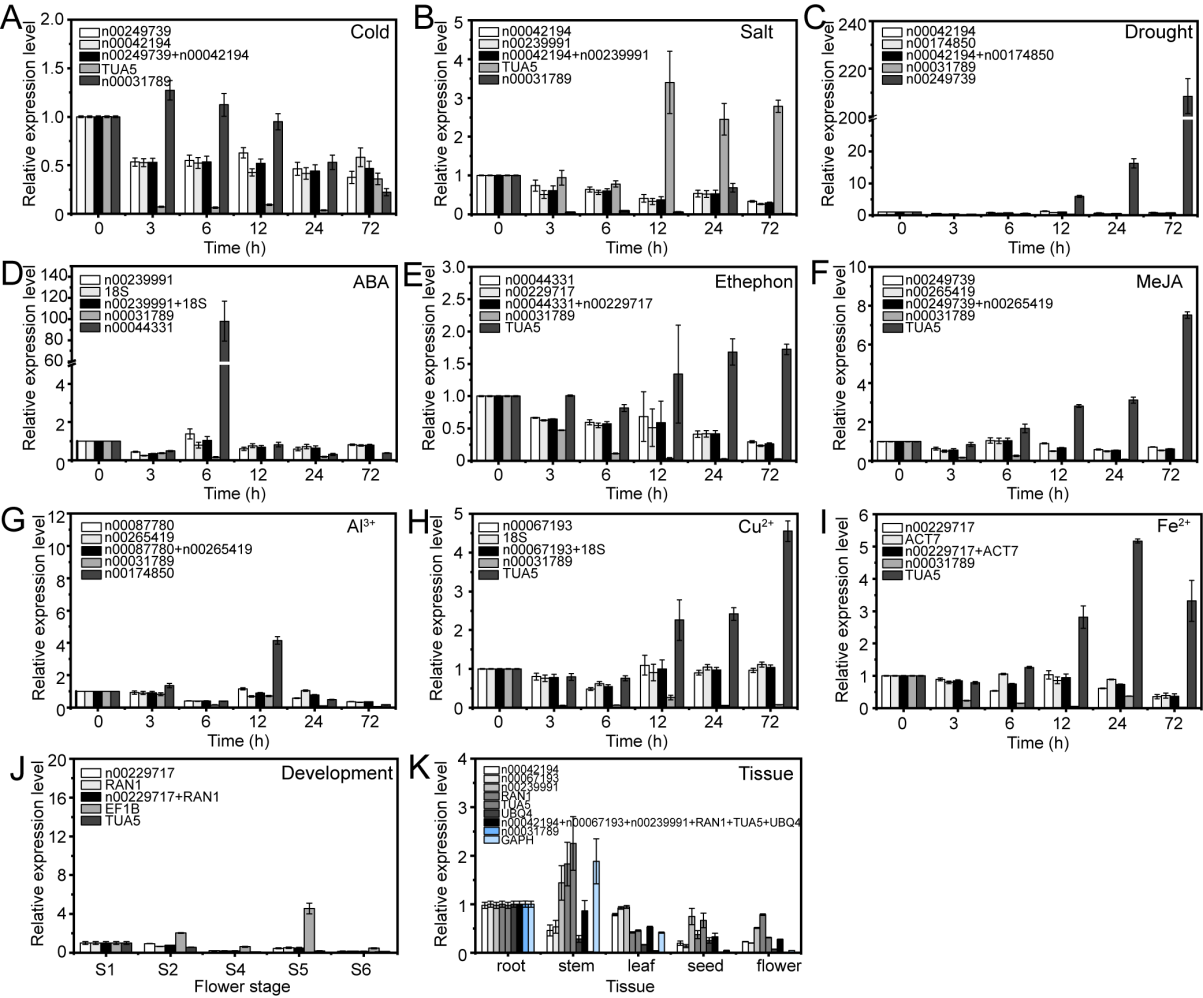
Relative expression levels of lnc00003036 under different experimental conditions, normalized using stable and unstable RGs. **(A)** Cold, **(B)** salt, and **(C)** drought stress; **(D)** ABA, **(E)** ethephon, **(F)** MeJA, **(G)** Al^3+^, **(H)** Cu^2+^, and **(I)** Fe^2+^ treatments; **(J)** flowering stage; and **(K)** different tissues.

**Figure 9:**
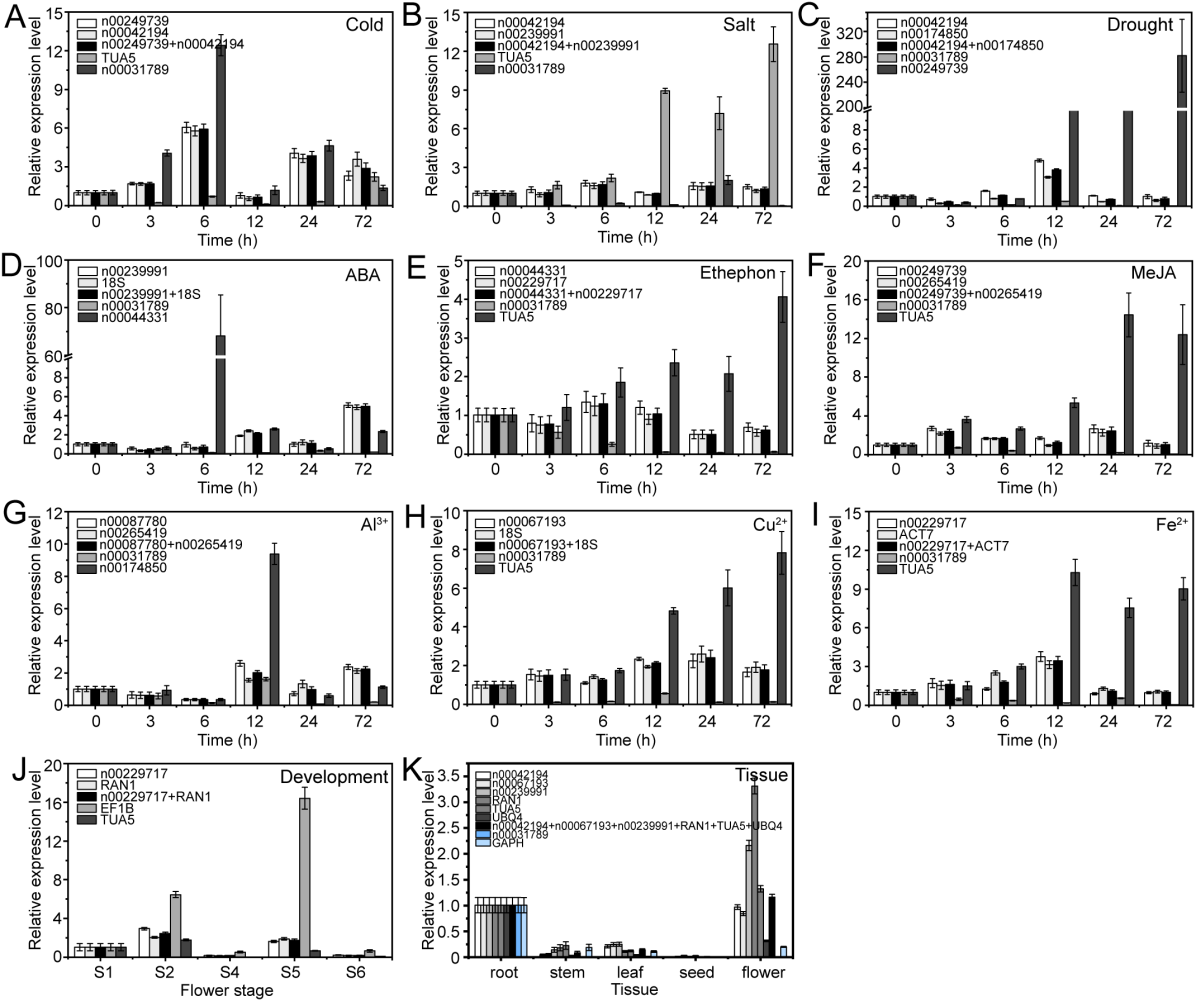
Relative expression levels of lnc00126603 under different experimental conditions, normalized using stable and unstable RGs. **(A)** Cold, **(B)** salt, and **(C)** drought stress; **(D)** ABA, **(E)** ethephon, **(F)** MeJA, **(G)** Al^3+^, **(H)** Cu^2+^, and **(I)** Fe^2+^ treatments; **(J)** flowering stage; and **(K)** different tissues.

**Figure 10:**
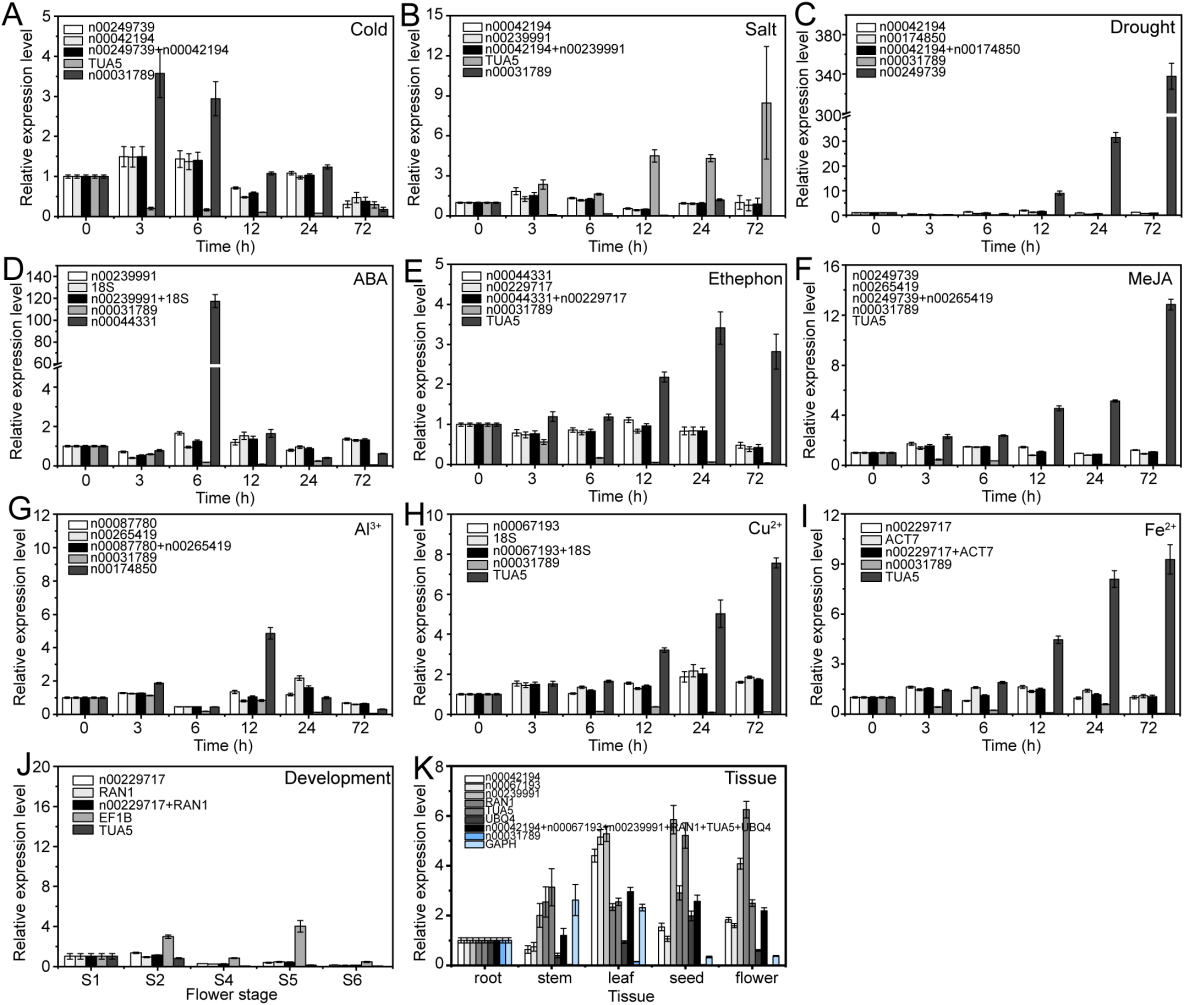
Relative expression levels of lnc00250780 under different experimental conditions normalized using stable and unstable RGs. **(A)** Cold, **(B)** salt, and **(C)** drought stress; **(D)** ABA, **(E)** ethephon, **(F)** MeJA, **(G)** Al^3+^, **(H)** Cu^2+^, and **(I)** Fe^2+^ treatments; **(J)** flowering stage; and **(K)** different tissues.

The expression patterns varied depending on whether the most stable or unstable RGs were used under specific experimental conditions (**Figures 8**–**10**). For example, under low-temperature conditions, the expression of lnc00003036 decreased when normalized using stable RGs (**Figure 8A**). In contrast, normalization with the unstable reference lnc00031789 resulted in an initial increase followed by a decrease, peaking at 3 hours (**Figure 8A**). When the unstable reference *TUA5* was used, the expression of lnc00003036 initially decreased and then increased (**Figure 8A**). Across various treatments, while the target genes displayed similar expression patterns when normalized with different stable RGs, their expression levels varied between treatments (**Figures 8**–**10**). For instance, during flower opening and senescence stages, lnc00126603 displayed a pattern of increase, decrease, increase, and decrease again when normalized with stable RGs such as lnc00229717, *RAN1*, and the combination of lnc00229717 and *RAN1*. However, using the unstable *EFIB* as an internal RG resulted in a similar trend for lnc00126603, but with an expression level during the S5 period that was eight times higher compared to normalization with stable RGs (**Figure 9J**). Overall, the choice of RGs for normalizing target gene expression can significantly impact the results. The incorrect selection of RGs may lead to inaccurate estimates of the relative expression of target genes.

## 4 Discussion

Gene expression analysis is essential for understanding gene function, particularly in biological research (Bourgeois et al., 2016; Torres-Rodríguez et al., 2024). Among the various methods available for detecting gene expression levels, qRT-PCR is a widely employed technique for examining gene expression patterns (Wei et al., 2020; Zhang et al., 2021b). However, achieving accurate qRT-PCR results can be challenging due to limitations in primer design flexibility and the lack of suitable RGs for standardizing gene expression. Previous studies have indicated that lncRNAs are generally expressed at lower levels compared to mRNAs (Wu et al., 2014; Chen et al., 2018). Consequently, using protein-coding genes as internal RGs for studying lncRNA expression may not accurately reflect their true expression levels. The use of common RGs without rigorous screening can compromise the accuracy of quantitative analyses and potentially lead to erroneous conclusions. Therefore, it is essential to meticulously screen RGs before their use to ensure the precision of qRT-PCR analyses (Huang et al., 2014; Wei et al., 2020; Zhang et al., 2021b; Wang et al., 2024a). Currently, there are no reports on lncRNA in *O. fragrans*. To enhance the accuracy of gene expression studies, we systematically screened for reliable internal RGs to normalize lncRNA expression across various tissues of *O. fragrans* and under different stress conditions. This approach aims to improve the reliability of gene expression studies and provide more accurate insights into gene function in this species.

In this study, the E values for the candidate RG primers ranged from 91.216% to 111.998%, with an R^2^ ≥ 0.973 (**Table 1**). These results indicate that the primers used for screening the RGs exhibit high accuracy, efficiency, and sensitivity. Additionally, the average Ct values of the candidate RGs ranged from 10.556 (*18S*) to 30.580 (lnc00174850) (**Figure 2**). Among these, lnc00031789 exhibited the greatest variation in Ct values (15.664 Ct), ranging from 22.301 to 37.965, while *18S* showed the smallest variation (3.820 Ct), with maximum and minimum Ct values of 12.496 and 8.676, respectively (**Figure 2**). These findings align with previous studies on species such as *C. fortunei* (Zhang et al., 2021a, b), gerbera flowers (*Gerbera hybrida*) (Li et al., 2022), dogbane (*Apocynum venetum*) (Li et al., 2024), tomato (*Solanum lycopersicum*) (Løvdal and Lillo, 2009), soybean (*Glycine soja*) (Liu et al., 2016), and arrowheads (*Sagittaria trifolia*) (Tang et al., 2023), where candidate RGs exhibited varying expression levels across different test materials. Although the expression level of *18S* was relatively stable across all samples, it was not consistent under different stress treatments or in different tissues (**Figure 2**). Therefore, the selection of appropriate RGs for normalizing lncRNA expression under specific experimental conditions is crucial.

We employed four commonly used algorithms (delta-Ct, geNorm, NormFinder, and BestKeeper) to evaluate and identify stable RGs. The results revealed that the top five genes selected by the different algorithms were generally consistent (**Supplementary Figure S1**). For example, under low-temperature stress, the algorithms consistently identified *GAPH*, lnc00249739, and lnc00265419 as relatively stable genes (**Supplementary Figure S1**). Similarly, under salt stress, abiotic stress, MeJA treatment, Fe^2+^ treatment, and across all samples, at least three of the top three genes identified by these algorithms were the same (**Supplementary Figures S1B, D, F, K, O**). However, the stable genes identified by different algorithms varied significantly under each experimental condition (**Figures 3**–**7**). For instance, under low-temperature stress, the delta-Ct and NormFinder algorithms identified lnc00042194 and *GAPH* as the most stable genes, whereas BestKeeper and geNorm identified lnc00239991 and lnc00249739, and lnc00265419 and lnc00249739, respectively (**Figures 3**–**6**). This variation is consistent with findings in other plant species (Vandesompele et al., 2002; Zhang et al., 2018, 2021a, b; Wang et al., 2024a). We speculate that these discrepancies arise from differences in the calculation methods used by the algorithms and their varying sensitivities to co-regulated candidate genes. Therefore, it is essential to comprehensively consider the results from these algorithms to select the most appropriate RGs for practical applications.

We employed the geometric mean of ranks method in conjunction with RefFinder to conduct a comprehensive analysis of RG stability. Based on the optimal number of RGs determined by geNorm analysis, we identified the most stable RG combinations. Notably, the results from the geometric mean of ranks method were consistent with those obtained from RefFinder across various treatments and tissues (**Figure 7**; **Supplementary Table S3**). Specifically, the most stable RG combinations identified under different conditions were as follows: lnc00249739 and lnc00042194 for cold stress; lnc00042194 and lnc00174850 for drought stress; lnc00239991 and lnc00042194 for salt stress; lnc00239991, lnc00042194, lnc00067193, and lnc00265419 for abiotic stress; lnc00265419 and lnc00249739 for MeJA treatment; lnc00229717 and lnc00044331 for ethephon treatment; lnc00265419 and lnc00239991 for hormone treatment; lnc00067193 and *18S* for Cu^2+^ treatment; lnc00239991 and lnc00067193 for metal ion treatments; lnc00229717 and *RAN1* for flower opening and senescence processes; lnc00239991, lnc00042194, lnc00067193, *TUA*, *UBQ4*, and *RAN1* for various tissues; and lnc00239991, lnc00042194, lnc00265419, and *UBQ4* for all samples (**Figure 7**; **Supplementary Table S3**). These findings suggest that the analysis conducted in this study is accurate. It is noteworthy that the optimal RG combinations varied across different treatments and tissues. Specifically, under ABA treatment, lnc00239991 and *18S* (instead of lnc00229717) were the most stable RGs; under Al^3+^ treatment, lnc00087780 and lnc00265419 (instead of lnc00249739) were optimal; and under Fe^2+^ treatment, lnc00229717 and *ACT7* (instead of lnc00249739) were the most stable RGs (**Figures 7E, I, K**; **Supplementary Table S3**). Similar variations in RG stability have been reported in other plants (Zhang et al., 2021a, b). We speculate that these differences may result from varying environmental conditions, tissue types, and treatment methods, which impact gene expression stability and lead to different RG combinations exhibiting optimal stability under distinct circumstances.

Although no single RG consistently emerged as the best choice across all experimental conditions, lnc00239991, lnc00042194, lnc00265419, and *UBQ4* were identified as good candidates for normalizing lncRNA expression in *O. fragrans*. Notably, *UBQ4* was identified as the optimal RG in a qRT-PCR study of rice seedling leaves under salt stress (Moraes et al., 2015) and for perennial ryegrass (*Lolium perenne*) under various abiotic stresses (Huang et al., 2014). In contrast, *UBQ10* was found to be the most unstable RG in sudangrass (*Sorghum sudanense*) under NaCl stress (Wang et al., 2024b). These discrepancies can be attributed to the specific expression patterns of RGs and their varying suitability across different conditions. Furthermore, the lncRNAs lnc00239991, lnc00042194, and lnc00265419 have rarely been reported as internal RGs in plants, highlighting the generally lower expression levels of lncRNAs. Therefore, selecting protein-coding genes as internal RGs to study lncRNA expression may not always accurately reflect their true expression levels of lncRNAs.

Numerous studies have demonstrated that selecting appropriate RGs is crucial for gene expression research using qRT-PCR technology. Inappropriate RG selection can lead to erroneous conclusions. In this study, we utilized both stable and unstable RGs to examine the expression patterns of target lncRNAs across various stress conditions, different tissues, and during flower opening and senescence. Our results showed that using inappropriate candidate RGs can lead to the underestimation or overestimation of lncRNA expression or misinterpretation of expression trends (**Figures 8**–**10**). This highlights the critical importance of selecting appropriate RGs for accurate molecular biological analyses.

## 5 Conclusions

In summary, this study represents the first systematic and comprehensive investigation into the selection and evaluation of 17 candidate RGs for normalizing lncRNA expression in *O. fragrans* under abiotic stress conditions, as well as hormone and metal ion treatments. Although no single RG was proved to be optimal across all experimental conditions, lnc00239991, lnc00042194, lnc00265419, and *UBQ4* emerged as reliable choices for lncRNA expression studies in *O. fragrans*. The findings facilitate accurate and thorough analysis of lncRNA expression across various conditions. This research provides a solid foundation for further studies exploring the roles of lncRNAs in growth and development, as well as their responses to abiotic stress, hormone treatments, and metal ion treatments. Furthermore, it provides valuable insights that could drive advancements in crop improvement and plant biotechnology by enhancing our understanding of lncRNA functions. Future research could explore the molecular mechanisms through which these lncRNAs influence plant stress responses, offering potential strategies for improving stress tolerance in *O. fragrans* and other crops.

## Data Availability

The datasets presented in this study can be found in online repositories. The names of the repository/repositories and accession number(s) can be found in the article/**Supplementary Material**.
