# Supplementary material for: Selection of suitable reference lncRNAs for gene expression analysis in Osmanthus fragrans under abiotic stresses, hormone treatments, and metal ion treatments
Source: Front Plant Sci. 2025 Jan 21;15:1492854. doi: 10.3389/fpls.2024.1492854 (PMC11790643; doi:10.3389/fpls.2024.1492854)
Supplement: Supplementary file 1 [file DataSheet1.docx]

Supplementary Material

# Supplementary Figures and Tables

## Supplementary Figures


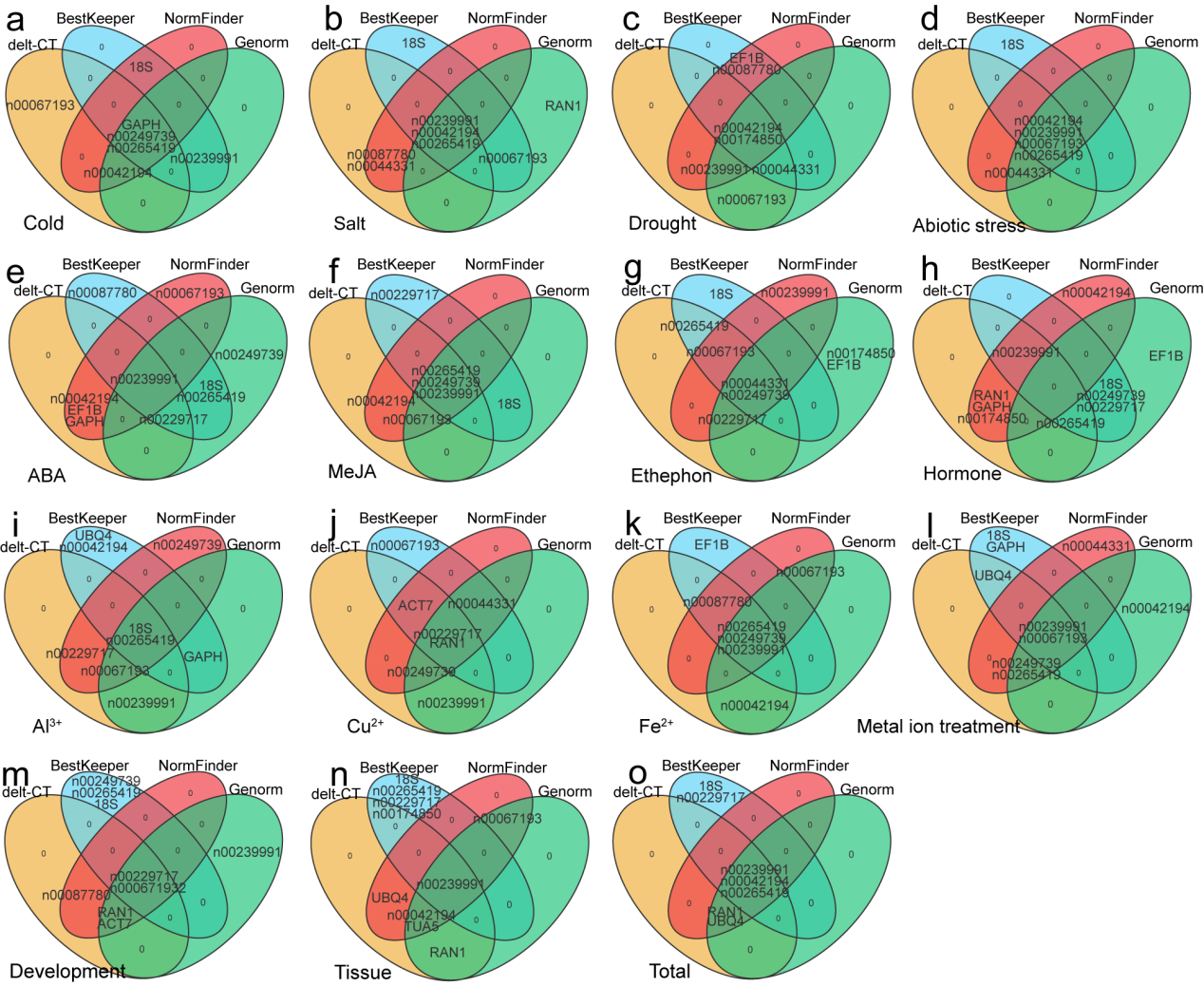
**Supplementary Figure 1.** The 5 most stable RGs indicated by delta-Ct analysis, geNorm, NormFinder, and BestKeeper. The blue, pink, green, and yellow circles each contain the 5 most stable RGs determined by BestKeeper, NormFinder, geNorm, and elta-Ct analysis, respectively. The genes in the overlapping area are those confirmed as the 5 most stable RGs by more than one algorithm.

## Supplementary Tables

**Table S1.** The expression levels of lncRNAs.

| LncRNA FPKM | S1 | S2 | S3 | S4 | S5 | S6 |
| --- | --- | --- | --- | --- | --- | --- |
| lnc_00031789 | 12.535 | 13.927 | 14.352 | 16.109 | 11.738 | 14.040 |
| lnc_00042194 | 85.425 | 77.610 | 68.251 | 69.473 | 68.199 | 65.176 |
| lnc_00044331 | 48.219 | 39.398 | 50.18 | 44.538 | 48.158 | 41.355 |
| lnc_00067193 | 60.367 | 54.624 | 63.376 | 67.381 | 63.366 | 51.326 |
| lnc_00087780 | 21.611 | 26.492 | 22.089 | 24.680 | 22.540 | 19.320 |
| lnc_00174850 | 22.126 | 22.784 | 29.431 | 27.795 | 29.898 | 27.089 |
| lnc_00229717 | 11.382 | 11.012 | 10.834 | 12.067 | 12.040 | 11.890 |
| lnc_00239991 | 27.517 | 26.969 | 26.105 | 25.394 | 21.119 | 20.907 |
| lnc_00249739 | 21.593 | 19.398 | 17.476 | 19.879 | 21.724 | 17.280 |
| lnc_00265419 | 367.995 | 344.906 | 398.901 | 390.969 | 346.185 | 345.834 |

**Table S2.** Statistics for the last two RGs in the stability rankings according to different evaluation methods.

|  | **Delta-Ct** | **GeNorm** | **NormFinder** | **BestKeeper** |
| --- | --- | --- | --- | --- |
| cold stress | *EF1B* | *EF1B* | *EF1B* | *TUA5* |
|  | n00031789 | n00031789 | n00031789 | n00031789 |
| salt stress | *TUA5* | *TUA5* | *TUA5* | *TUA5* |
|  | n00031789 | n00031789 | n00031789 | n00031789 |
| PEG stress | n00031789 | n00031789 | n00031789 | n00031789 |
|  | n00249739 | n00249739 | n00249739 | n00249739 |
| abiotic stress | n00031789 | n00031789 | n00031789 | n00031789 |
|  | n00249739 | n00249739 | n00249739 | n00249739 |
| ABA treatment | n00031789 | n00031789 | n00031789 | n00031789 |
|  | n00044331 | n00044331 | n00044331 | n00044331 |
| MeJA treatment | *TUA5* | *TUA5* | *TUA5* | *TUA5* |
|  | n00031789 | n00031789 | n00031789 | n00031789 |
| ethephon treatment | *TUA5* | *TUA5* | *TUA5* | *TUA5* |
|  | n00031789 | n00031789 | n00031789 | n00031789 |
| hormone treatment | n00031789 | n00031789 | n00031789 | n00044331 |
|  | n00044331 | n00044331 | n00044331 | n00031789 |
| Cu^2+^ treatment | *TUA5* | *TUA5* | *TUA5* | *TUA5* |
|  | n00031789 | n00031789 | n00031789 | n00031789 |
| Fe^2+^ treatment | *TUA5* | *TUA5* | *TUA5* | *TUA5* |
|  | n00031789 | n00031789 | n00031789 | n00031789 |
| Al^3+^ treatment | n00174850 | n00174850 | n00174850 | n00174850 |
|  | n00031789 | n00031789 | n00031789 | n00031789 |
| metal ion treatment | *TUA5* | *TUA5* | *TUA5* | *TUA5* |
|  | n00031789 | n00031789 | n00031789 | n00031789 |
| tissue | *18S* | *18S* | *18S* | *GAPH* |
|  | n00031789 | n00031789 | n00031789 | n00031789 |
| flowering stage | *TUA5* | *TUA5* | *TUA5* | *TUA5* |
|  | *EF1B* | *EF1B* | *EF1B* | *EF1B* |
| all samples | n00249739 | n00249739 | n00249739 | *EF1B* |
|  | n00031789 | n00031789 | n00031789 | n00031789 |

**Table S3.** Comprehensive ranking of stability of 17 candidate RGs using RefFinder.

|  | Cold stress | Salt stress | PEG stress | Abiotic stress | ABA treatment | MeJA treatment | Ethephon treatment | Hormone treatment |
| --- | --- | --- | --- | --- | --- | --- | --- | --- |
| 1 | n00249739 | n00239991 | n00042194 | n00042194 | n00239991 | n00265419 | n00229717 | n00265419 |
| 2 | n00042194 | n00042194 | n00174850 | n00239991 | *18S* | n00249739 | n00044331 | n00239991 |
| 3 | *GAPH* | n00265419 | n00239991 | n00067193 | n00265419 | *18S* | n00249739 | *18S* |
| 4 | n00265419 | n00087780 | *EF1B* | n00265419 | n00229717 | n00239991 | n00067193 | *RAN1* |
| 5 | n00239991 | n00067193 | n00067193 | n00044331 | n00042194 | n00067193 | n00174850 | n00249739 |
| 6 | *18S* | *18S* | n00044331 | *18S* | *EF1B* | n00042194 | *EF1B* | *GAPH* |
| 7 | n00067193 | n00044331 | n00087780 | *UBQ4* | *GAPH* | *GAPH* | n00265419 | n00174850 |
| 8 | *UBQ4* | *RAN1* | *RAN1* | *GAPH* | n00249739 | *RAN1* | *18S* | n00229717 |
| 9 | *RAN1* | *EF1B* | n00265419 | *RAN1* | n00087780 | *UBQ4* | n00239991 | *EF1B* |
| 10 | n00229717 | n00174850 | *18S* | n00087780 | n00174850 | *ACT7* | *GAPH* | n00042194 |
| 11 | *ACT7* | *UBQ4* | n00229717 | n00229717 | n00067193 | n00229717 | n00042194 | n00067193 |
| 12 | n00044331 | n00229717 | *UBQ4* | *EF1B* | *ACT7* | n00087780 | *ACT7* | n00087780 |
| 13 | n00087780 | *GAPH* | *GAPH* | n00174850 | *RAN1* | n00174850 | n00087780 | *UBQ4* |
| 14 | n00174850 | *ACT7* | *ACT7* | *ACT7* | *TUA5* | *EF1B* | *UBQ4* | *ACT7* |
| 15 | *EF1B* | n00249739 | *TUA5* | *TUA5* | *UBQ4* | n00044331 | *RAN1* | *TUA5* |
| 16 | *TUA5* | *TUA5* | n00031789 | n00031789 | n00031789 | *TUA5* | *TUA5* | n00031789 |
| 17 | n00031789 | n00031789 | n00249739 | n00249739 | n00044331 | n00031789 | n00031789 | n00044331 |
|  | Al^3+^ treatment | Cu^2+^ treatment | Fe^2+^ treatment | Metal ion treatment | Tissue | Flowering stage | All samples |  |
| 1 | n00087780 | n00067193 | n00229717 | n00239991 | n00042194 | n00229717 | n00239991 |  |
| 2 | n00265419 | *18S* | *ACT7* | n00067193 | n00239991 | *RAN1* | n00042194 |  |
| 3 | n00239991 | n00265419 | n00249739 | n00249739 | n00067193 | n00239991 | n00265419 |  |
| 4 | n00249739 | n00239991 | n00239991 | *UBQ4* | *UBQ4* | n00067193 | *UBQ4* |  |
| 5 | n00042194 | *UBQ4* | *RAN1* | n00265419 | *TUA5* | n00249739 | *RAN1* |  |
| 6 | n00067193 | *GAPH* | n00044331 | n00042194 | *RAN1* | *ACT7* | n00067193 |  |
| 7 | *UBQ4* | n00229717 | n00067193 | *ACT7* | n00265419 | n00087780 | *18S* |  |
| 8 | *EF1B* | n00042194 | *UBQ4* | n00044331 | n00249739 | n00265419 | *ACT7* |  |
| 9 | n00044331 | n00249739 | *GAPH* | *GAPH* | *18S* | n00042194 | n00229717 |  |
| 10 | *ACT7* | *RAN1* | n00087780 | *18S* | *ACT7* | *18S* | n00174850 |  |
| 11 | *GAPH* | *EF1B* | n00265419 | n00229717 | n00174850 | n00031789 | *GAPH* |  |
| 12 | *18S* | n00174850 | n00042194 | *RAN1* | n00229717 | n00044331 | n00087780 |  |
| 13 | *RAN1* | *ACT7* | *EF1B* | n00087780 | n00044331 | *UBQ4* | *TUA5* |  |
| 14 | n00229717 | n00044331 | n00174850 | *EF1B* | *EF1B* | *GAPH* | n00044331 |  |
| 15 | *TUA5* | n00087780 | *18S* | n00174850 | n00087780 | n00174850 | *EF1B* |  |
| 16 | n00174850 | *TUA5* | *TUA5* | *TUA5* | *GAPH* | *TUA5* | n00249739 |  |
| 17 | n00031789 | n00031789 | n00031789 | n00031789 | n00031789 | *EF1B* | n00239991 |  |

n, lnc.
